# Does Back Pain Go on Holiday in the Summer?

**DOI:** 10.3390/jfmk7040075

**Published:** 2022-09-26

**Authors:** Federico Roggio, Giuseppe Musumeci

**Affiliations:** 1 Department of Biomedical and Biotechnological Sciences, Section of Anatomy, Histology and Movement Science, School of Medicine, University of Catania, Via S. Sofia n°97, 95123 Catania, Italy; 2 Sport and Exercise Sciences Research Unit, Department of Psychology, Educational Science and Human Movement, University of Palermo, Via Giovanni Pascoli 6, 90144 Palermo, Italy; 3 Research Center on Motor Activities (CRAM), University of Catania, Via S. Sofia n°97, 95123 Catania, Italy

Back pain is one of the leading causes of disability among adults worldwide. According to the Global Burden of Diseases, it is the fourth leading cause among adults aged 25–49 and the sixth for those aged 50–74 [1]. It can comprise both radicular and nociceptive pain and causes functional limitations to daily activities, commonly in the neck or low back areas. It is defined as a self-limited condition: treatments are not necessary, and it resolves in 4–8 weeks in more than 50% of patients [2]. More than 60% of those with low back pain will experience it again within one year of onset, exemplifying its chronicity [3]. However, pain is not just a neurological signal from A-delta and C fibers; it also comprises the psychosocial sphere, e.g., emotional and cognitive aspects. Besides its physiologic nature, it is well-established that different conditions play a key role in back pain onset in terms of lifestyle. Sedentary behavior, diet disorders, smoking, and the absence of physical activity represent the primary factors; however, secondary factors, such as sleep problems, depression, anxiety, work issues, air conditioning, muscle contractures, or awkward posture during daily activities (e.g., driving or working), can trigger its onset. 

Seasonality represents a variation in which a specific condition experiences regular changes that recur yearly—such as the weather. In this discussion, we consider the seasonal changes in health aspects, such as the morbidity of cardiovascular diseases and the occurrence of metabolic syndrome, which show a higher prevalence during the winter [4]. Seasonality in health conditions is difficult to investigate because following a sample of patients for one year may be challenging for both individuals and researchers. In recent years, different studies have investigated the seasonality of some musculoskeletal conditions, such as rheumatoid arthritis, osteoarthritis, back pain, fibromyalgia, and ankle and knee pain, unconventionally: through Google Trends searches [5]. Even if this approach does not represent a true disease expression, it reflects what users are interested in searching through Google and what they are probably experiencing at the moment of that search. According to Ciaffi and colleagues [5], back pain appears to have seasonal fluctuations. Specifically, the summer represents the season with a lower volume of searches about back pain and other concerns. The researchers observed only a relative search volume on that topic and did not correlate it with any real patient information; however, based on this concept, we wondered if specific conditions promote a diminished back pain experience during the summer. 

During the winter season, individuals dedicate more time to work and less to leisure time, inclement weather may hinder sports activities, and usually, individuals gain weight due to a more caloric diet. Prolonged working time and hindered physical activity possibilities promote a sedentary behavior correlated with back pain onset. Furthermore, work stress can exacerbate depression and anxiety [6]—mental health comorbidities associated with back pain onset [7]. Meanwhile, the warm season brings pleasant weather that stimulates individuals to increase physical activity and leisure time with friends. Besides the well-known positive effects of physical activity on back pain reduction, the summer season supports individuals’ positive moods, impacting psychological and hormonal changes. Sunshine plays a key role by stimulating the skin under two specific conditions, i.e., serotonin and vitamin D levels, both season-related. Serotonin levels have been demonstrated to increase during prolonged exposition to the sun thanks to the retinoraphe tract [8] and possibly to the sun exposition of the skin, suggesting that the skin may be involved in its production and bioregulation [9]. Moreover, pharmacological treatments of serotonin levels are widely used in managing psychological pathologies and, recently, in the management of back pain [10]. Finally, the sunshine vitamin, vitamin D, is essential for the musculoskeletal system. During the summer, the sun’s exposition increases, and so there is a great production of vitamin D. On the contrary, sun exposure during most of the winter does not lead to any production of vitamin D [11]. Even if a clear relationship has not yet been demonstrated, vitamin D deficiency has been observed to be associated with low back pain [12]. 

This discussion may pique the interest of those involved in the seasonality changes in human health. Although the back has a “good memory”, we can answer our hypothesis by asserting that back pain during the summer is probably reduced by increased levels of serotonin, physical activity, and vitamin D. Therefore, we would recommend outdoor physical activity, sunbathing, long walks, and swimming while using adequate sunscreen during the summer to send back pain on holiday.

## Data Availability

Not applicable.
